# Effect of Vitamin D Analogue on Rosuvastatin-Induced Myopathy in Wistar Rats

**DOI:** 10.1155/2020/4704825

**Published:** 2020-03-31

**Authors:** Bharti Chogtu, Balaji Ommurugan, Sereen R. Thomson, Sneha G. Kalthur

**Affiliations:** ^1^Department of Pharmacology, Kasturba Medical College, Manipal, Manipal Academy of Higher Education, Manipal, Karnataka 576104, India; ^2^BioPharma Solutions, Chennai, Tamil Nadu, India; ^3^APCER Life Sciences, Ahmedabad, Gujarat, India; ^4^Department of Anatomy, Kasturba Medical College, Manipal, Manipal Academy of Higher Education, Manipal, Karnataka 576104, India

## Abstract

**Objectives:**

Statin-induced myopathy is one of the major causes of poor adherence and discontinuation of this medication. There are contrary results regarding association of vitamin D insufficiency with statin-induced myopathy. This study was done to determine the effect of the vitamin D3 analogue alfacalcidol on Rosuvastatin-induced myopathy in rats. *Methodology*. Animals were divided into six groups with 6 rats in each group. Groups I and II acted as controls, Group III and Group IV were administered Rosuvastatin 120 mg/kg/day and 160 mg/kg/day, Groups V and VI were administered alfacalcidol 0.1 *μ*g/kg/day in addition to Rosuvastatin 120 mg/kg/day and 160 mg/kg/day, respectively. All drugs were administered orally for 15 days. Plasma creatine kinase (CK) levels were estimated on day 10 and day 15. Animals were sacrificed and muscles were sent for histopathological examination.

**Results:**

On day 10, Groups V and VI showed a statistically significant increase in plasma CK levels as compared to the control (*p* < 0.001) and were significantly lower (*p* < 0.001) as compared to Groups III and IV, respectively. However, on day 15, plasma CK levels in Groups V and VI were comparable to those of control groups with a nonsignificant difference (*p* > 0.05). On comparing the histology, Groups V and VI showed a significant difference as compared to statin-only groups (Groups III and IV) as there were signs of regeneration, less splitting, and fragmentation of muscle fibres.

**Conclusion:**

The present study shows that the vitamin D analogue alfacalcidol prevents statin-induced myopathy. The serum CK levels are comparable to the control group on day 15 of vitamin D administration.

## 1. Introduction

Hydroxy-methyl-glutaryl Coenzyme A (HMG CoA) reductase inhibitors or statins offer one of the most effective strategies for reducing cardiovascular diseases. They are mainstay in management of dyslipidemia, coronary artery disease, hypertension, and stroke. Beyond lipid lowering, the pleiotropic actions of statins include improvement in endothelial action, antioxidant and anti-inflammatory action, and stabilization of atherosclerotic plaques [1]. These actions are mediated by inhibiting the HMG CoA reductase which in turn alters cell membrane stability and mitochondrial function and reduces cholesterol in the cell membrane [2]. Statins are considered to be safe and tolerable drugs. However, 40 to 75% of patients on statins discontinue the therapy within 1–2 years of initiation [3]. The nonadherence to statins increases the risk of acute cardiovascular events. The nonadherence and discontinuation of statins is multifactorial, and one of the major reasons is statin induced muscle-related side effects which range from mild myalgia to fatal rhabdomyolysis.

The various statins being prescribed are atorvastatin, pravastatin, rosuvastatin, and fluvastatin. The effect of different statins on lipid profile is dose dependent, and Rosuvastatin, a relatively newer member of the statin family of drugs, has a superior effect in improving lipid profile as observed in Asian population [4]. Rosuvastatin is linked with high incidence of muscle-related adverse effects in comparison to other statins [5]. Incidences of myopathy have been reported with higher doses of Rosuvastatin. Product labelling in Europe highlights this risk at high doses of statins (40 mg once a day) [6].

Low level of vitamin D is associated with muscle weakness and myopathy [7]. Studies have linked vitamin D insufficiency to statin myopathy as well. In a cross-sectional study, in individuals with 25OH vitamin D less than 15 ng/ml, use of statin was associated with myopathy as compared to nonstatin users [8]. Similarly, a study in Pennsylvania in which patients were categorized based on vitamin D levels and vitamin D deficiency at statin initiation was associated with development of statin-induced myopathy subsequently [9]. However, few studies did not find any relationship between plasma vitamin D levels and risk of muscle-related adverse effects in statin users [10]. A retrospective study conducted in Israel in 6708 patients reported that there is no association between low 25 hydroxy vitamin D levels and statin-induced myalgia [11].

Controversy regarding whether vitamin D insufficiency leads to statin-induced myalgia or statins contribute to vitamin D deficiency persists [12]. With this background, the present study aimed at evaluating the effect of vitamin D analogue alfacalcidol on rosuvastatin-induced myopathy in rats and also compared the effects of vitamin D analogue on different doses of statins to produce statin-induced myopathy.

## 2. Methodology

After obtaining clearance from the Institutional Animal Ethics Committee, the study was initiated. Wistar rats aged 6 to 8 weeks were acclimatised for 6 days prior to the study. Animals were kept in well-illuminated rooms with a 12-hour light/dark cycle and temperature and humidity controlled. Pelleted rodent diet and drinking water was freely available to them.

A total of 36 rats were included in the study, and animals were divided into 6 groups, with each group consisting of 6 rats. Group I was administered 0.5% hydroxypropyl methylcellulose (control 1), group II 0.5% polyoxyl 40 hydrogenated castor oil (control 2), group III Rosuvastatin 160 mg/kg/day dissolved in 0.5% hydroxypropyl methylcellulose, group IV Rosuvastatin 120 mg/kg/day dissolved in 0.5% hydroxypropyl methylcellulose, group V alfacalcidol 0.1 *μ*g/kg/day dissolved in polyoxyl 40 hydrogenated castor oil + Rosuvastatin 120 mg/kg/day dissolved in 0.5% hydroxypropyl methylcellulose, and group VI alfacalcidol 0.1 *μ*g/kg/day dissolved in polyoxyl 40 hydrogenated castor oil + Rosuvastatin 160 mg/kg/day dissolved in 0.5% hydroxypropyl methylcellulose. The dose of Rosuvastatin is as per the earlier study [13].

All the drugs were administered orally daily for 15 days. The animals were observed and weighed every week. Weight of the animals at the end of the study was recorded. Blood was withdrawn on day 10 and day 15 for plasma chemistry.

  Plasma chemistry: blood samples were collected to evaluate plasma creatine kinase levels using a spectrophotometer.
  Histology: the animals were sacrificed, and the skeletal muscle tissue soleus was taken out and sent for histological examination. Tissues were fixed in buffered 10% formalin, processed to wax blocks, and then sectioned and stained with hematoxylin and eosin for examination by light microscopy. During the histopathological examination of the muscle sections, necrosis was graded minimal to severe depending on the number of fibres affected.
  Statistics: the comparison of plasma creatine kinase levels between the groups was done using analysis of variance (ANOVA) followed by post hoc Tuckey's test. Comparison of histological features between the groups was done using the Kruskal–Wallis test. *p* value <0.05 was taken as significant.

## 3. Results and Discussion

**Figure 1:**
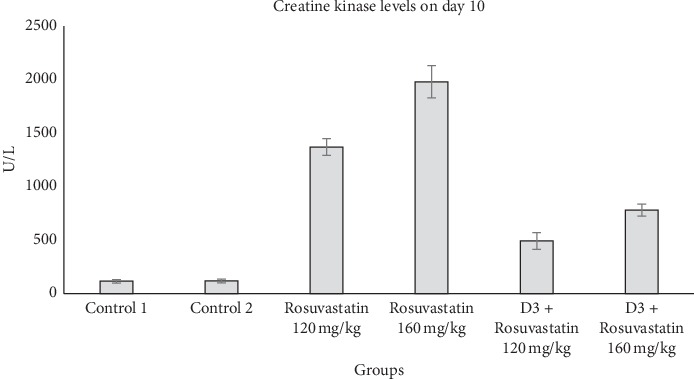
Plasma creatine kinase (CK) levels in different groups on day 10. Values are expressed in mean ± SEM.

The creatine kinase (CK) levels were estimated on day 10 and day 15. The creatine kinase levels in different groups on day 10 are shown in Figure 1. Using ANOVA, a significant difference (*p* < 0.001) was seen between the groups. Post hoc analysis did not show any significant difference between the CK levels of the two control groups. Compared to the control groups, significantly increased CK levels were seen in statin 120 mg/kg (*p* < 0.001) and statin 160 mg/kg (*p* < 0.001) groups. The statin 120 mg/kg + vitamin D3 group showed a significantly decreased (*p* < 0.001) CK levels compared to the statin 120 mg/kg group. Similarly, the statin 160 mg/kg + vitamin D3 group showed decreased levels of CK levels compared to the statin 160 mg/kg group which was statistically significant (*p* < 0.001). However, CK levels in vitamin D3 + Rosuvastatin 120 mg/kg and vitamin D3 + Rosuvastatin 160 mg/kg were significantly (*p* < 0.001) higher than that of the control groups on day 10.

**Figure 2:**
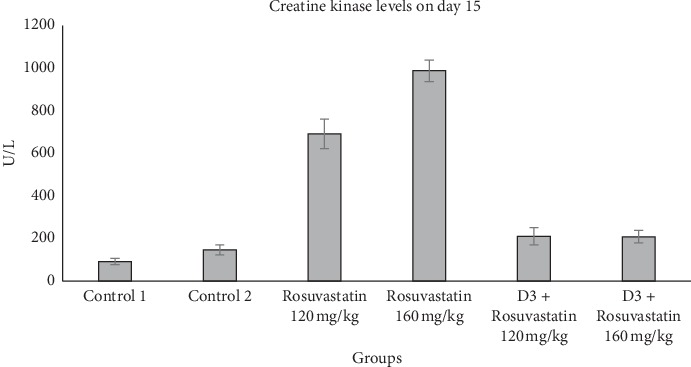
Plasma creatine kinase levels in different groups on day 15. Values are expressed in mean ± SEM.

Day 15 CK values showed a significant difference between the groups by using ANOVA. CK levels increased significantly (*p* < 0.001) in Rosuvastatin 120 mg/kg and Rosuvastatin 160 mg/kg groups. Also, CK levels were significantly higher (*p* < 0.001) in Rosuvastatin 120 mg/kg and Rosuvastatin 160 mg/kg groups as compared to vitamin D3 + Rosuvastatin 120 mg/kg and vitamin D3 + Rosuvastatin 160 mg/kg groups, respectively. Also, CK levels in vitamin D3 + Rosuvastatin 120 mg/kg and vitamin D3 + Rosuvastatin 160 mg/kg groups did not differ significantly (*p* > 0.05) from that of the control groups on day 15, as shown in Figure 2.

**Figure 3:**
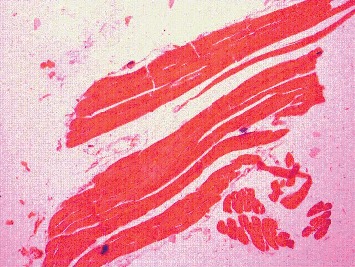
Histology of muscle fibres in the control group.

**Figure 4:**
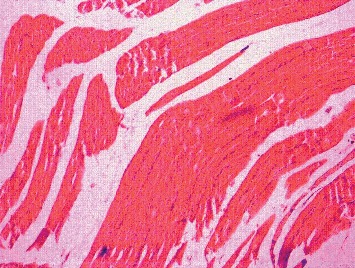
Histology of muscle fibres in the Rosuvastatin 160 mg/kg group.

**Figure 5:**
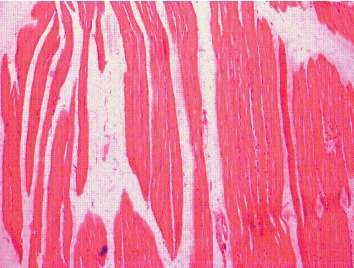
Histology of muscle fibres in the Rosuvastatin 160 mg/kg + vitamin D3 group.

The Kruskal–Wallis test showed that there is a statistically significant difference in histology scoring between the different groups with chi-square (*χ*2) of 35.59 and *p* value <0.001.  Figure 3 shows histology of muscle fibres in the control group which revealed normal structure of skeletal muscle. Cylindrical muscle fibres with elongated nuclei are seen peripherally. Rosuvastatin 160 mg/kg resulted in vacuolization and fragmentation/splitting of fibres. Nuclei appear rounded and concentrated in the centre. Inflammatory cellular infiltration was present through the endomysium and some muscle fibres are rounded (Figure 4). Vitamin D3 + Rosuvastatin 160 mg/kg showed muscle regeneration in the form of hypercellularity with many nuclei and satellite cells. There was less edema and striations, and peripheral nuclei are faintly observed in this high-dose statin + vitamin D3 (Figure 5).

## 4. Discussion

Statins have revolutionized the management of patients with cardiovascular disorders. However, the associated adverse effects with statins can be a cause of poor patient compliance. Myalgia, myopathy, rhabdomyolysis, hepatotoxicity, headache, rash, and gastrointestinal side effects are encountered in patients on statins [14].

CK is physiologically present in the plasma and is used as a marker in myopathic processes [15]. A rise in the CK activity is a result of muscle ﬁbre destruction after mechanical trauma, toxic injury, or alteration of enzymatic or structural proteins. CK levels are used to monitor the safe use of statins. CK elevation varies within disorders, with increases that may range from 2- to 100-fold the reference value. By inhibiting the membrane cholesterol synthesis, statins modify membrane excitability in tissues like skeletal muscles. ATP production in mitochondria is dependent on coenzyme Q10 [16]. Statins inhibit the vital step in the synthesis of coenzyme Q10 and have a role in statin-induced myopathy. Thus, statins trigger skeletal muscle apoptosis and myopathy. Some of the agencies state that statins should be discontinued immediately if CK levels are more than ten times of the upper limit of normal [17].

Statins inhibit CYP3A4, which has 25-hydroxylase activity in vitro [18]. In case of vitamin D deficiency, CYP3A4 may potentially get shunted for vitamin D hydroxylation, to maintain the levels of 25OH vitamin D, reducing availability of CYP3A4 for statin metabolism. This increases serum statin levels and causes statin toxicity. This hypothesis supports that supplementation of vitamin D can prevent statin-induced myopathy. However, no association between vitamin D levels and statin-induced myopathy has been reported by some authors. A study in a cardiology outpatient clinic in Groningen could not find any relationship between vitamin D status and statin-related myopathy [19]. Another study conducted in 129 patients could not find any association between vitamin D levels and statin-associated myalgia in which authors reiterated that there is a need to further examine the role of vitamin D deficiency in statin-associated myopathy [20].

The present study was done to determine the effect of combination of vitamin D analogue with statin and statin alone on CK levels and muscle fibre histology in wistar rats. The CK levels were measured on day 10 and day 15. On both the days, the CK levels in vitamin D3 + Rosuvastatin 120 mg/kg and vitamin D3 + Rosuvastatin 160 mg/kg was significantly lower than the Rosuvastatin 120 mg/kg and Rosuvastatin 160 mg/kg, respectively. On the 10th day, CK levels in vitamin D3 + Rosuvastatin 120 mg/kg and vitamin D3 + Rosuvastatin 160 mg/kg were significantly (*p* < 0.001) greater than the control groups. CK levels in vitamin D3 + Rosuvastatin 120 mg/kg and vitamin D3 + Rosuvastatin 160 mg/kg groups did not differ significantly (*p* > 0.05) from that of the control groups on the 15^th^ day. This implies that as the duration of intake of vitamin D increases, the CK levels start falling and even become comparable to control. A significant decrease in CK levels was seen both in 120 mg/kg and 160 mg/kg groups when alfacalcidol was added. So the protective effect of vitamin D at high dose was observed in this study.

In statin users with vitamin D levels less than 20 ng/ml, supplementation of vitamin D can improve statin tolerance [21]. Patients with 25OH vitamin D less than 50 nmol/L have four times increased risk of developing statin-induced myopathy. Individuals homozygous for specific genetic polymorphism in the vitamin D receptor have increased risk of developing muscle-related symptoms [22]. In 200 patients taking atorvastatin, myopathy increases with increase in dose was demonstrated in a pilot study [23].

On the contrary, findings of a study done in a large cohort did not show any association between low-serum vitamin D levels and CK elevation or statin-induced myalgia [11]. In a case series of 11 patients, 8 patients had statin-induced myalgia and authors even concluded that screening of vitamin D should be performed in patients with statin-induced myalgia [24]. Thus, vitamin D can be considered as a modifiable risk factor in patients with statin-induced myopathy.

Even supplemental vitamin D enhances the effect of statins like atorvastatin on lipid profile [25]. Vitamin D levels >30 nmol/l were required for atorvastatin to reduce lipid levels, as in lower doses of vitamin D the hypolipidemic effect of statins was not seen [26].

Histology of skeletal muscle fibres was compared between the groups. Normal histology was seen in hydroxypropyl methylcellulose and castor oil group (two control groups). In statin-only group, degenerated fibres were seen which appeared swollen. Loss of striations and nuclei concentrated in the centre, peculiarities of fibre damage, were seen. More vacuolization and fragmentation were seen in groups with higher doses of statin (Rosuvastatin 160 mg/kg). However, in the combination group (Rosuvastatin 160 mg/kg + vitamin D3 group), the regeneration in the form of hypercellularity with multiple nuclei was seen. The histology of this group was comparable to that of the control group. Cellular infiltration and splitting of fibres are characteristic features of myotoxicity. Alterations in the form of splitting, focal degeneration of myofibrils, and collagen deposition between the myocytes were observed with Rosuvastatin at a high therapeutic dose for 4 and 12 weeks [27]. Statins cause myocyte damage by altering cholesterol content in cell membranes, and muscle cells have unique segregation of lipids and proteins making it susceptible to damage by statins [28]. Decreased cholesterol modulates fluidity and alters the function of Na^+^ K^+^ pumps which could lead to degeneration of organelles and thus damaging cells [29]. Other changes like muscle fibre splitting occur because of compensatory hypertrophy and cellular infiltration and are secondary to release of mediators during myocyte degradation [30]. Most of the studies have shown that type I fibres are less sensitive to statin-induced myopathy as compared to the type II fibres. Studies have put forth that statins at low doses show toxic effect in type II fibres and at high doses type I fibres are affected [31]. Atorvastatin 10 mg/kg and 40 mg/kg mainly affected type II fibres and spared type I fibres in rats. However, type I fibres were mainly affected by atorvastatin at a dose of 80 mg/kg [31]. This observation can be correlated with clinical studies where statin-induced myopathy has been shown to be dose related. In the present study, high doses of statins were used and toxic effects were seen in soleus which predominantly has type I fibres. Also, in a study by Obayashi et al., Cerivastatin-induced myotoxicity was seen only in type I fibres and not in type II fibres in 344 young male rats [32].

This study supports that vitamin D can prevent statin-induced myopathy. The limitations of this study are that electron microscopy of tissues and other tissue parameters have not been done which could have given a deeper insight into the mechanism of statins at subcellular levels.

## 5. Conclusion

The present study shows that the vitamin D analogue alfacalcidol prevents statin-induced myopathy and even reverses the histological changes induced in muscles by statins in animal models. Studies with longer follow-up and studying other histological parameters will help in further confirming the role of vitamin D in statin-induced myopathy and its exact mechanistic role. Confirmatory studies can further shed light on the potential use of Vitamin D in patients who are to be started on statins.

## Data Availability

Data used to support the findings of this study are available from the corresponding author upon request.
